# Electrochemical Oxidation of Para-Aminophenol With Rare Earth Doped Lead Dioxide Electrodes: Kinetics Modeling and Mechanism

**DOI:** 10.3389/fchem.2019.00382

**Published:** 2019-06-11

**Authors:** Mili Weng, Xihe Yu

**Affiliations:** School of Environmental and Resource Sciences, Zhejiang A & F University, Hangzhou, China

**Keywords:** electrochemical oxidation, rare earth, doped lead dioxide electrodes, kinetics modeling, mechanism

## Abstract

In this study, La and Ce doped PbO_2_ electrodes were prepared and the characteristic of the electrodes were discussed with the help of structure analysis. The catalytic effects of the doped electrodes were explored through the degradation of para-aminophenol wastewater. The results showed that the para-aminophenol removal was 96.96%, 89.34%, and 77.55% after 180 min treatment with Ce-PbO_2_, La-PbO_2_, and PbO_2_, respectively. The para-aminophenol enhanced degradation mechanism was discussed with rare earth element doping electrodes and a kinetic model was established based on radical reactions mechanism with genetic algorithm (GA) calculation. The reaction constants of these electrodes were calculated and the results showed that the reaction constant of Ce-PbO_2_ electrode was the highest, which indicated that Ce-PbO_2_ electrode could have a better treatment effect. The EE/O was used as the index of energy consumption efficiency and the results were calculated and compared. This paper could provide basic data and technique reference of the prediction the oxidation reaction process of different electrodes for the electrochemical oxidation application in wastewater treatment.

## Introduction

Para-aminophenol (PAP) is the primary material of paracetamol industry, which was widely used in a number of countries (Bloomfield, 2002; Bahrami and Salehabadi, 2014). Due to the characteristic of the pharmacy production process, the PAP would be contained in the wastewater. If this kind wastewater was discharged directly into water environment, there would affect human's health (Hallman and Tarloff, 2000; Li et al., 2004; Harmon et al., 2005; Khodaveisi et al., 2015). Therefore, the degradation and mineralization of PAP wastewater and relative pharmaceutical wastewater has received a great concern in the field of environment.

As one of advanced oxidation processes (AOPs), electrochemical oxidation could remove bio-refractory organic pollutants in wastewater and has the advantages of relatively high treatment efficiency with easy operation (Velegraki et al., 2010; Chen et al., 2014; Xia et al., 2015a). Electrode materials, such as boron-doped diamond, platinum, IrO_2_, RuO_2_, and PbO_2_ and carbon relative materials (Arapoglou et al., 2003; Zhuo et al., 2011; Li et al., 2014; Wang et al., 2019), play an important role in electrochemical reaction. The β-PbO_2_ electrode and rare earth element doped β-PbO_2_ electrode were widely investigated due to the easy preparation technology and excellent degradation effect (Aquino et al., 2014; Mukimin et al., 2015; Xia et al., 2015b; Dai et al., 2016). However, the study of the reactions kinetic model on the doped and undoped PbO_2_ electrodes is still limited.

In this study, PAP was selected as the model pollutant and the degradation of PAP wastewater was studied with the electrodes of PbO_2_, La doped PbO_2_ (La-PbO_2_), and Ce doped PbO_2_ (Ce-PbO_2_). A kinetic model was established based on genetic algorithm (GA) calculation and used to quantify the performance of electrode materials. This paper could provide basic data and theoretical support for PAP wastewater treatment by electrochemical oxidation.

## Experimental

### Chemicals

PAP was selected as the model pollutant and obtained from Shanghai J&K Chemical Reagent (China) Co., Ltd. (purity 99.5%, wt%). The structure formula and general characteristics of PAP were given in Table 1. Other chemicals were purchased from Aladdin Reagent (China) Co., Ltd. All the other reagents were of analytical grad.

**Table 1:** General characteristics of PAP.

**Name**	**4-Aminophenol**
Chemical structure	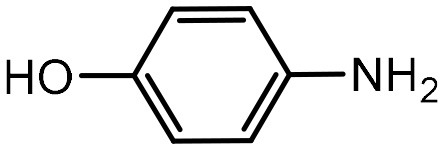
Water solubility	1.5 g/100 mL (20°C)
CAS number	123-30-8

### Electrode Preparation

The detail process for the preparation and characteristic of the electrodes has been fully described in previous literatures (Weng et al., 2013; Dai et al., 2014; Weng and Pei, 2016). Briefly, the electrode included three layers: the Ti substrate inner layer, thermally deposited Sn-SbOx intermediate layer, and the electrodeposited α-PbO_2_ and β-PbO_2_ outer layer. When La-PbO_2_ and Ce-PbO_2_ electrodes were prepared, certain amounts of La or Ce nitrate were added to the acid solution for the electro-deposition of β-PbO_2_. Compared with undoped PbO_2_ electrode, the surface and structure analysis results of doped PbO_2_ showed that the electrodes after doped with rare earth could effectively improve the crystallinity of PbO_2_ crystal on electrode surface. The tinier grain crystals on the doped electrodes could promote the conductivity, stability, and catalytic activity of electrode, which would be potential widely applied in dye wastewater pollution control in application filed.

### Analysis Methods

The PAP concentration was analyzed by high performance liquid chromatography (Agilent 1200, USA) with C18 column and UV detector. The UV detector wavelength was set at 220 nm. The injection volume was 10 μl. The mobile phase was methanol and H_3_PO_4_ solution (1‰) and the ratio of methanol with H_3_PO_4_ solution was 22:78 (v/v). The flow rate was 0.4 ml min^−1^. The crystal structure was investigated by X-ray diffraction (XRD) instrument with CuKα radiating (Rigaku D/MAX 2500PC, Japan) under condition of 40 kV voltage and 300 mA tube current. The continuous scan mode was used and the scan speed was 4°·min^−1^ with the range of 10°~90°.

The average current efficiency (ACE) was calculated by chemical oxygen demand (COD) and current data at time intervals (Comninellis and Pulgarin, 1991).

(1) $$\text{ACE}=\frac{{\text{COD}}_{0}-{\text{COD}}_{\text{t}}}{8\text{It}}\text{FV}$$

Where: COD_0_ and COD_t_ are the CODs (in O, g·L^−1^) of samples at different time (s), respectively. F is the faraday constant (96487 C·mol^−1^), V is the volume of the electrolyte (L), I is the electrolysis current (A), and t is the reaction time (s).

### Experimental Procedure

PAP wastewater degradation experiment was performed in a 250 ml customize reactor. During PAP degradation, samples were taken at presumed intervals to determine the concentration of PAP, COD, and total organic carbon (TOC). All tests were run three times. The results for each experimental condition were the means of the three experiments and the relative standard deviations (RSD) were <5%.

### Modeling Approach

To establish model, the rate constants were calculated with the degradation data of PAP concentration. The minimum value of residual between experimental data and modeling data as the objective function was calculated by GA calculation (Goldberg, 1989; Ndiritu and Daniell, 2001).

The objective function (OF) was listed as follows:

(2) $$\text{OF}=\sqrt{\frac{1}{\text{n}-1}{\sum_{\text{i}=1}^{\text{n}}\left(\frac{{\text{C}}_{\text{data},\text{i}}-{\text{C}}_{\text{model},\text{i}}}{{\text{C}}_{\text{data},\text{i}}}\right)}^{2}}$$

Where: OF = objective function, dimensionless, n = number of data points, unitless

C_data,i_ = measured concentration of data point i, mmol L^−1^,

C_model,i_ = predicted concentration of data point i, mmol L^−1^

All the reaction constants (k) were calculated by the GA method.

## Results and Discussion

### PAP Degradation With Different Electrodes

**Figure 1:**
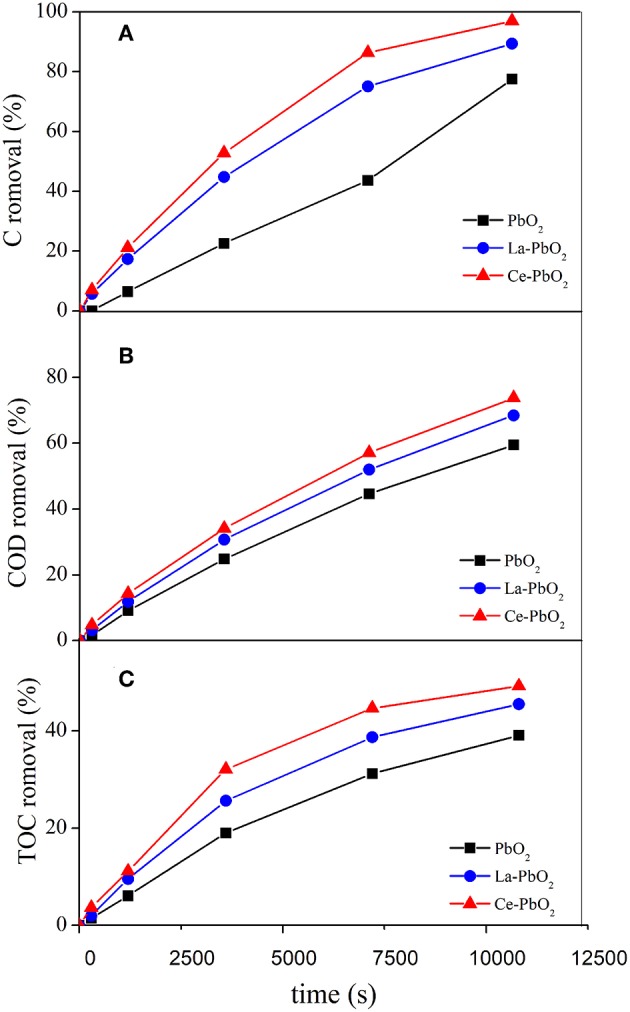
Effect of different electrodes on PAP degradation: **(A)** Concentration Removal, **(B)** COD Removal, **(C)** TOC Removal.

PbO_2_ electrode, La-PbO_2_ electrode and Ce-PbO_2_ electrode were applied for the degradation of PAP wastewater on the condition of electrolyte concentration Na_2_SO_4_ 0.1 mol·L^−1^, PAP concentration 500 mg·L^−1^ and current density 70 mA·cm^−2^. The degradation results were shown in Figure 1.

The PAP removal with Ce-PbO_2_ electrode was 96.96%, which is higher than the removal with La-PbO_2_ (89.34%) and PbO_2_ electrodes (77.55%) after 3 h treatment. The COD and TOC removal after 3 h for these three electrodes was 73.79, 68.49, 59.49%, and 49.23, 45.47, 39.08%, respectively. And these results were showed in Figures 1A–C, respectively. Under the same conditions, the ACE of the La-PbO_2_ and Ce-PbO_2_ electrode were higher 8.35 and 11.69% than that of undoped electrode (42.05%). The results demonstrated that the catalytic activity of La-PbO_2_ and Ce-PbO_2_ were improved degradation of PAP effectively. Also, the Ce-PbO_2_ electrode displayed a higher removal efficiency and faster mineralization than La-PbO_2_ and PbO_2_ electrodes for PAP degradation.

### Materials Characterization

**Figure 2:**
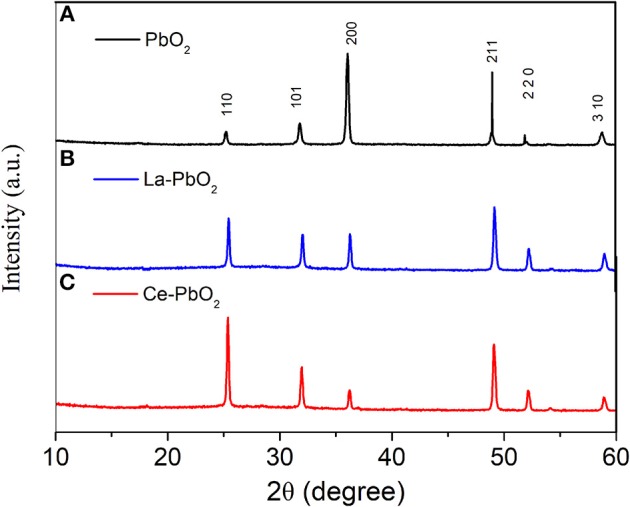
XRD of different electrodes: **(A)** PbO_2_, **(B)** La-PbO_2_, **(C)** Ce-PbO_2_.

Figure 2 showed the XRD patterns of different electrodes. Compared with PbO_2_, La and Ce doping would cause the change of crystal orientation of β-PbO_2_. After doped, the lattice plane of (110) was enhanced, which would influence the catalytic activity for organics degradation. The SEM results showed that after doped, the electrode surface had a tinier crystal structure and higher degree of crystallinity (Weng et al., 2013), which means that the addition of Ce could effectively improve the crystallinity of PbO_2_ crystal on electrode surface, and thus could increase the efficiency of PAP degradation.

### Kinetics Model Study

#### The Key Reaction in Electrochemical Oxidation System

**Figure 3:**
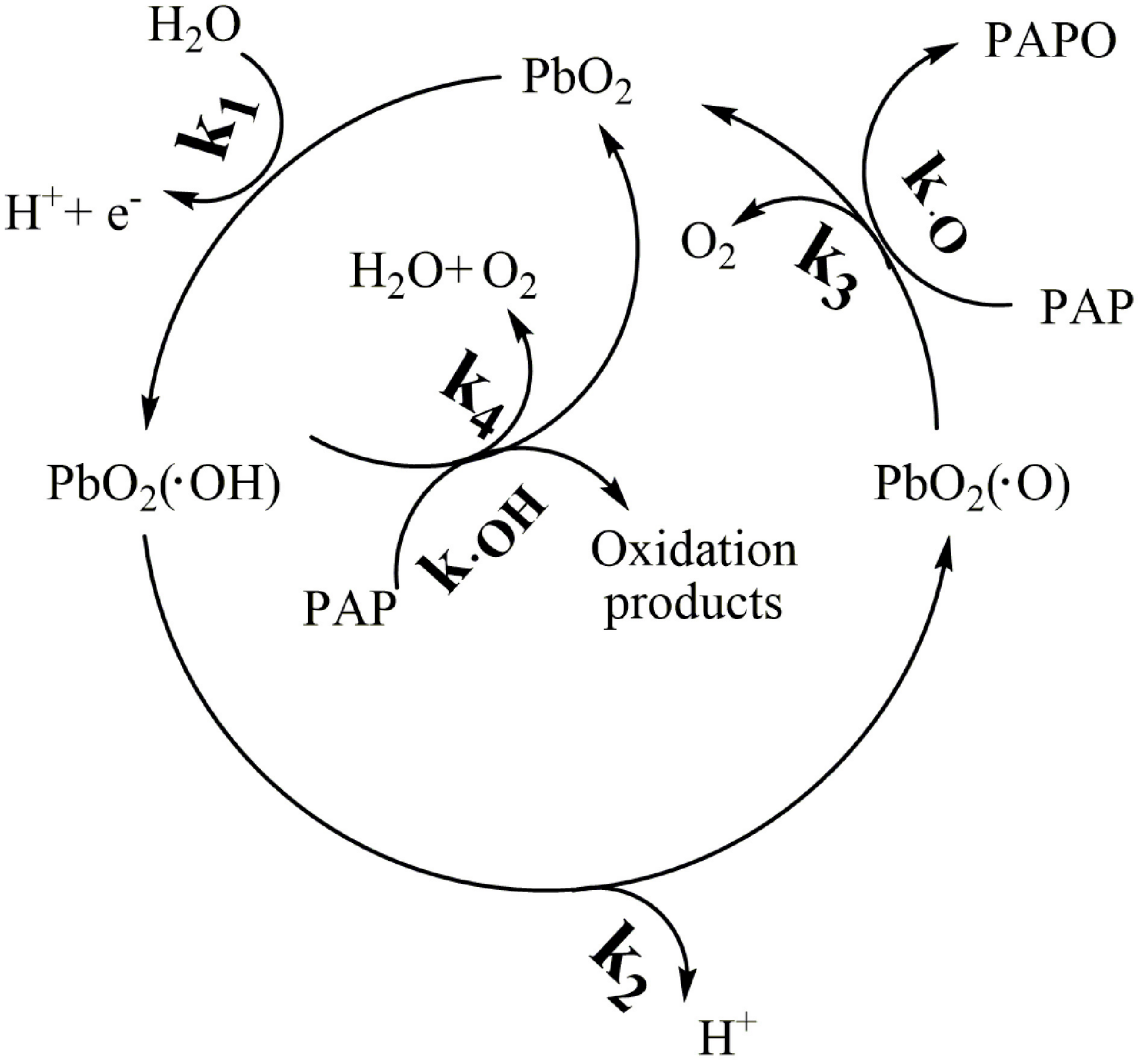
Mechanism analysis of electrochemical oxidation PAP.

A generalized scheme of the electrochemical degradation of PAP with PbO_2_ electrode was shown in Figure 3. Firstly (No. 1, Table 2), H_2_O was decomposed on the electrode surface to yield adsorbed hydroxyl radical PbO_2_ (•OH). Secondly (No. 2, Table 2), hydroxyl radical on the oxide electrode could both react with PAP and form the lattice oxide of PbO_2_ (•O). Thus, there are two kinds of “active oxygen” could be existed on the PbO_2_ electrode surface: physisorption “active oxygen” PbO_2_ (•OH) and chemisorbed “active oxygen” PbO_2_ (•O) (Comminellis, 1994).

**Table 2:** Mainly elementary reactions involved in electrochemical oxidation system.

**No**	**Reactions**	**Rate Constants, mol L^**−1**^s^**−1**^**
1	H_2_O +PbO_2_ → PbO_2_(**·**OH)+H^+^+e^−^	k_1_
2	PbO_2_(**·**OH) → PbO_2_(**·**O)+H^+^+e^−^	k_2_
3	PbO_2_(**·**O) → PbO_2_ + 0.5O_2_	k_3_
4	PbO_2_(**·**O)+PAP → PAP(O) + PbO_2_	k_**·**O_
5	PbO_2_(**·**OH)+ PAP → oxidation products	k_**·**OH_
6	PbO_2_(**·**OH)+ PbO_2_(**·**OH) → 2PbO_2_+0.5O_2_+H_2_O	k_4_
7	**·**OH +HC_3_^−^ → CO_3_^−^·+H_2_O	k_HCO_3_^2−^_ = 8.5 × 10^6^
8	**·**OH +CO_3_^2−^ → CO_3_^−^·+ OH_−_	k_CO_3_^2−^_ = 3.9 × 10^8^
9	H_2_CO_3_ ⇌ H^+^+ HCO_3_^−^	pKa_1_ = 6.36
10	HCO_3_ ⇌ H^+^ + CO_3_^2−^	pKa_2_ = 10.25
11	H_2_O ⇌ H^+^ + OH^−^	pKa_3_ = 15.7

Due to the instability, the chemisorbed “active oxygen” PbO_2_(•O) would decompose according to the equation (No. 3, Table 2). The PbO_2_ (•O) would participate in the reaction of oxidation PAP, as was listed in the equation (No. 4, Table 2), while PbO_2_(•OH) complete fast reaction of oxidation PAP and mineralization them completely, according to No. 5 in Table 2 (Buxton et al., 1988; Simond et al., 1996). The hydroxyl radicals also might interact and the reaction constant of k_4_ means the interaction of hydroxyl radicals (No. 6, Table 2). The ionization balances and carbonate quenching equation were considered in our system list in Table 2.

From the No. 4 and No. 5 in Table 2, the ordinary differential equation (ODE) of PAP degradation rate with the time was deduced as follows, which included PAP reaction with PbO_2_ (•OH) and PbO_2_ (•O):

(3) $$\frac{\text{d }[\text{PAP​​}]}{\text{dt}}=-{\text{k}}_{•\text{OH}}[{\text{PbO}}_{2}(•\text{OH})][\text{PAP}]\;-{\text{k}}_{•\text{O}}[\text{Pb}(•\text{O})][\text{PAP}]$$

From the No. 1, 2, 5, 6, 7, and 8 in Table 2, the ODE of PbO_2_ (•OH) reaction rate variation with the time was studied, which included both yielding and decaying of PbO_2_(•OH):

(4) $$\begin{array}{l} \frac{\text{d }[\text{Pb}(\text{HO}•)]}{\text{dt}}={\text{k}}_{1}[{\text{H}}_{2}\text{O}]-{(\text{k}}_{2}+{\text{k}}_{4})[{\text{PbO}}_{2}(•\text{OH})]\\ \;-{\text{k}}_{•\text{OH}}[{\text{PbO}}_{2}(•\text{OH})][\text{PAP}]\\ \;-{\text{k}}_{{{\text{HCO}}_{3}}^{2-}}[{\text{PbO}}_{2}(•\text{OH})][{{\text{HCO}}_{3}}^{-}]\\ \;-{\text{k}}_{{{\text{CO}}_{3}}^{2-}}[{\text{PbO}}_{2}(•\text{OH})][{{\text{CO}}_{3}}^{2-}] \end{array}$$

From the No. 2, No. 3 and No. 4 in Table 2, the ODE of PbO_2_ (•O) reaction rate variation with the time was deduced, which included yielding and decaying of PbO_2_ (•O):

(5) $$\begin{array}{l} \frac{\text{d }[\text{Pb}(•\text{O})]}{\text{dt}}={\text{k}}_{2}[{\text{PbO}}_{2}(•\text{OH})]\;-{{\text{k}}_{\text{O}}}_{\cdot }[{\text{PbO}}_{2}(•\text{O})][\text{PAP}]\\ \;-{\text{k}}_{3}[{\text{PbO}}_{2}(•\text{O})] \end{array}$$

The ODE of HCO_3_^−^ and CO_3_^2−^ termination the reaction with the time was deduced, including the key reaction No. 7 and No. 8 in Table 2:

(6) $$\frac{\text{d }[{{\text{HCO}}_{3}}^{-}]}{\text{dt}}=-{\text{k}}_{{{\text{HCO}}_{3}}^{2-}}[{\text{PbO}}_{2}(•\text{OH})][{{\text{ HCO}}_{3}}^{-}]$$

(7) $$\frac{\text{d }\left[{{\text{CO}}_{3}}^{2-}\right]}{\text{dt}}=-{\text{k}}_{{{\text{CO}}_{3}}^{2-}}[{\text{PbO}}_{2}(•\text{OH})][{{\text{CO}}_{3}}^{2-}\text{​​}]$$

These ODEs were calculated, as were listed in Table 3. The reaction constants of k_1_, k_2_, and k_3_ represent the speed of PbO_2_(•OH), PbO_2_(•O) formation and PbO_2_(•O) decomposition, which were determined by the properties of electrode materials on a certain external condition. The larger value of k_1_ means that the production of HO• and degradation PAP efficient would be enhanced. The larger of k_2_ means that the larger probability of physisorbed •OH could transfer to PbO_2_(•O), which would react with PAP relatively slowly and competed with oxygen evolution from PbO_2_(•O) (represented by k_3_). As the concentration of hydroxyl radicals is very little, so the interaction of hydroxyl radicals (k_4_) could be ignored. The k_O_ and k_HO_ represent the speed of PAP reaction with “active oxygen.” These reaction constants were calculated by least square method and GA.

**Table 3:** The ODEs of the reaction kinetic model.

$\frac{\text{d }[\text{ PAP }]\;}{\text{dt}}=-{\text{k}}_{•\text{OH}}\;[{\text{PbO}}_{2}(•\text{OH})]\;[\text{ PAP }]\;-{\text{k}}_{\text{O}•}\;[\text{ Pb}(•\text{O})]\;[\text{ PAP }]$	(1)
$\frac{\text{d }[\text{ Pb}(•\text{OH})\;]\;}{\text{dt}}={\text{k}}_{1}\;[{\text{ H}}_{2}\text{O }]{-\text{k}}_{•\text{OH}}\;[{\text{ PbO}}_{2}(•\text{OH})]\;[\text{ PAP }]{\;-(\text{k}}_{2}+{\text{k}}_{4})[{\text{ PbO}}_{2}(•\text{OH})]{\;-\text{k}}_{{{\text{HCO}}_{3}}^{2-}}\;[{\text{ PbO}}_{2}(•\text{OH})\;]\;[{{\text{ HCO}}_{3}}^{-}\;]{\;-\text{k}}_{{{\text{CO}}_{3}}^{2-}}\;[{\text{ PbO}}_{2}(•\text{OH})\;]\;[{{\text{ CO}}_{3}}^{2-}\;]$	(2)
$\frac{\text{d }[\text{ Pb}(•\text{O})\;]\;}{\text{dt}}={\text{k}}_{2}\;[{\text{ PbO}}_{2}(•\text{OH})]{-\text{k}}_{•\text{O}}\;[{\text{ PbO}}_{2}(•\text{O})]\;[\text{ PAP }]{-\text{k}}_{3}\;[{\text{ PbO}}_{2}(•\text{O})]\;$	(3)
$\frac{\text{d }[{{\text{ HCO}}_{3}}^{-}\;]\;}{\text{dt}}={\text{- k}}_{{{\text{HCO}}_{3}}^{2-}}\;[{\text{ PbO}}_{2}(•\text{OH})\;]\;[{{\text{ HCO}}_{3}}^{-}\;]\;$	(4)
$\frac{\text{d}\left[{\text{CO}}_{3}2^{-}\right]}{\text{dt}}={-\text{k}}_{{\text{CO}}_{3}2^{-}}\;[{\text{ PbO}}_{2}(•\text{OH})\;]\;[{\text{ CO}}_{3}2^{-}\;]\;$	(5)

#### Rate Constants Calculated and Modeling

The model assumption as follows:

1. Solution in reaction system mixed completely, where species concentration uniform.
2. Reaction temperature was constant.
3. The O_2_ evolution of physisorbed “active oxygen” PbO_2_ (•OH) was ignored; The O_2_ evolution of chemisorbed “active oxygen” PbO_2_(•O) were considered. The PbO_2_ (•OH) and PbO_2_ (•O) were the main radical reaction with PAP in the solution.

**Figure 4:**
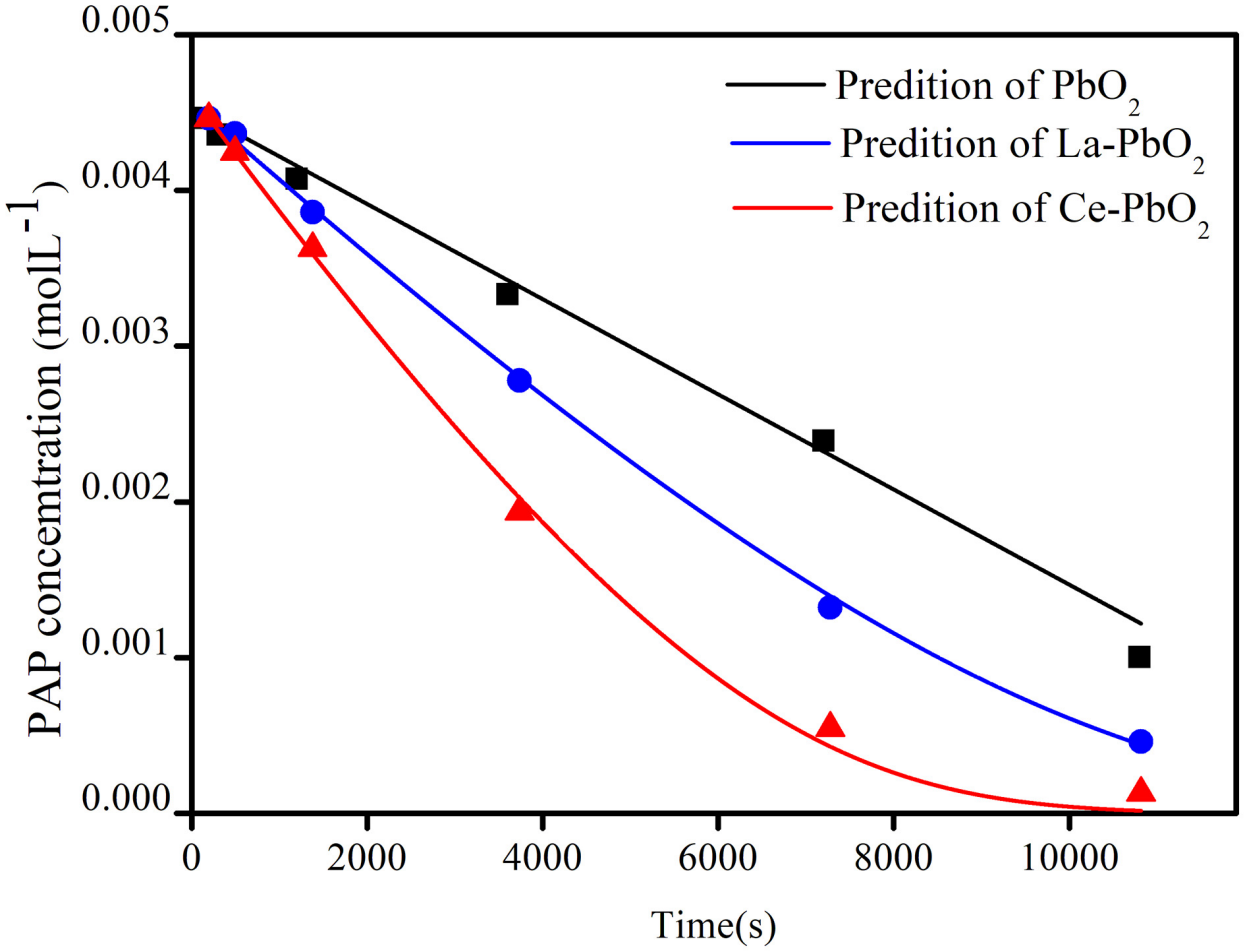
Modeling results of PAP degradation.

Figure 4 showed the comparison between the predicted and experimental decomposition of PAP under condition of PAP initial concentration 500 mg▪L^−1^, Na_2_SO_4_ 0.1 mol▪L^−1^ and current density 70 mA▪cm^−2^. The kinetic model was established by least square method and GA. The pseudo reaction constants k_▪O_ was much less than k_▪OH_, which indicated that the activity of physisorbed “active oxygen” is much stronger than chemisorbed “active oxygen.”

#### Kinetics and Mechanism Analysis

Compared with PbO_2_ electrode (a), the La doped PbO_2_ electrode (b) and Ce doped PbO_2_ electrode(c) would lead to increase active points on crystal plane (Feng et al., 2008), which would lead to the increase of k_1_ and decrease of k_2_ and k_3_. After comparing k_1_ of undoped and doped PbO_2_ electrodes, it could be found that Ce-PbO_2_ had a greater k_1_ value, which indicated PbO_2_(•OH) was easier produced from electrode material of Ce-PbO_2_. Comparing k_2_ and k_3_ of doped PbO_2_ electrodes, it could be found that Ce-PbO_2_ had a smaller k_2_ and k_3_ value, which indicated the speed of physisorbed “active oxygen” transfer to crystals and lattice oxygen releasing of superoxide was weaker. Therefore, the catalytic efficiency of the three electrodes is Ce-PbO_2_ > La-PbO_2_ > PbO_2_.

The intermediates during the PAP degradation were analyzed. Kinds of intermediates were detected at time intervals, including maleic acid, acetic acid, formic acid, NO_2_^−^ and NO_3_^−^. The results showed that the degradation of PAP would open loop into small molecule acid, such as maleic acid, acetic acid, formic acid on the condition of PbO_2_ (•OH) effective. Then kinds of small molecule acids would be degraded into to CO_2_ and H_2_O in the process of electrochemical oxidation.

#### EE/O Calculation

The electrical efficiency per log order (EE/O) is one of the best conceptions to describe energy utilization, which could be calculated by the established model. The EE/O calculation using equation (Crittenden et al., 2012; Butcher, 2016):

(8) $$\text{EE/O}=\frac{\text{P}\times \text{t}}{\text{V}\times {\text{lg}(\text{C}}_{0}{\text{/C}}_{\text{t}})}$$

**Figure 5:**
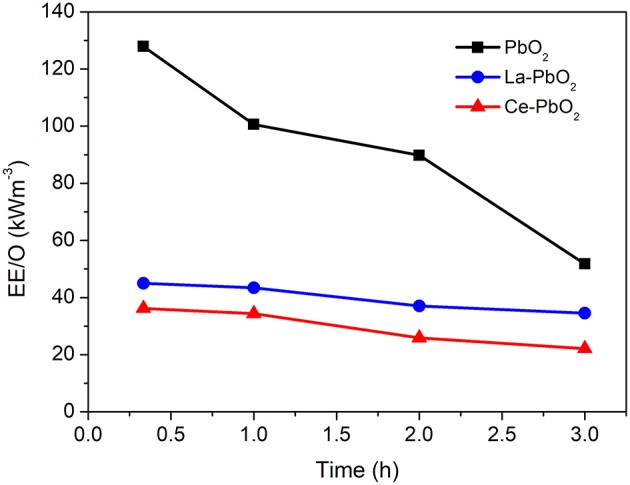
EE/O of different electrodes.

Where: EE/O = the electrical efficiency per log order reduction (kWh m^−3^);

P = the average power output (kW), t is the electrolysis time (h);

V = the wastewater volume (m^3^);

C_0_ = the initial PAP concentration (mg L^−1^);

C_t_ = the PAP concentration at different time (mg L^−1^);

The results of EE/O at time intervals were showed in Figure 5. The final EE/O of PbO_2_, La-PbO_2_, and Ce-PbO_2_ were 51.79 kWh m^−3^, 34.56 kWh m^−3^, 22.22 kWh m^−3^. The EE/O of Ce-PbO_2_ was <34% of La-PbO_2_ and 55% of PbO_2_.

## Conclusions

The Ce-PbO_2_ electrode was successful prepared and used for PAP wastewater treatment. It was found that the novel Ce doped electrode is much more energy saving than La-PbO_2_ and PbO_2_. The EE/O of Ce-PbO_2_ was 34% less than that of La-PbO_2_ and 55% of PbO_2_. A kinetic model was established base on GA calculation, which could help us evaluate the catalytic efficiency of doped PbO_2_ electrode and predicted the reaction process on the surface of the electrode. The main advantage of this paper is to give a method for the energy consumption analysis based on pseudo element reaction calculation. This paper could provide basic data and technique reference of the prediction the oxidation reaction process of different electrodes for the electrochemical oxidation application in wastewater treatment.

## Author Contributions

MW designed and co-carried the experiment, performed the analysis and wrote the manuscript. XY co-carried the experiment.

### Conflict of Interest Statement

The authors declare that the research was conducted in the absence of any commercial or financial relationships that could be construed as a potential conflict of interest.
